# Objective assessment of laparoscopic targeting skills using a Short-Time Power of Difference (STPOD) method

**DOI:** 10.1007/s11548-022-02622-2

**Published:** 2022-04-15

**Authors:** Shinji Ohtake, Kazuhide Makiyama, Daisuke Yamashita, Tomoyuki Tatenuma, Masahiro Yao

**Affiliations:** 1 Department of Urology, Yokohama City University Graduate School of Medicine, 3-9 Fukuura, Kanazawa-ku, Yokohama, Kanagawa 236-0004 Japan; 2 Department of Urology, Yokohama Sakae Kyosai Hospital, Yokohama, 247-8581 Japan

**Keywords:** Surgical skills, Laparoscopic surgery, Surgical training, Short-Time Power of Difference (STPOD), Motion smoothness, Objective assessment

## Abstract

**Purpose:**

To ensure that the use of surgical training tools results in improvement of surgical skills, it is necessary to be able to measure and assess surgeons’ skills. We established the Short-Time Power of Difference (STPOD) method as an evaluation tool for evaluating targeting technique. The STPOD method evaluates the distance from the actual movement of the forceps to the shortest linear path between two points in a short time period. We examined the effectiveness of the STPOD method as a new forceps kinematic analysis.

**Methods:**

Six residents were categorized as novices and six urologists as experts. All participants performed box trainer training and LapPASS^®^ Simulator training. During the procedure, objective scores (time, distance, and STPOD) were recorded. STPOD (Power) evaluated motion smoothness and STPOD (Stop) evaluated the stop time of the forceps.

**Results:**

STPOD (Stop) on the right side of the experts was significantly lower than that of the novices in the box trainer. Furthermore, there were significant differences in the distances of left side and STPOD (Power) between the experts and the novices in the simulator. In the correlation of parameters between the box trainer and the simulator, time showed the strongest correlation, STPOD (Power) and distance showed a mild correlation.

**Conclusion:**

We showed the construct validity of STPOD (Power) and STPOD (Stop) using both the box trainer and the simulator. This method is a good evaluation tool for assessing a physician’s skill; however, there are much more complex motions that are performed in actual surgery. Future studies are needed to focus on evaluation in an environment closer to actual surgery and comparing with other existing methods.

## Introduction

To ensure patient safety, it is desirable for surgeons to practice surgical procedures before performing them. In addition, in order to improve their skill levels and shorten learning curves and procedure times, surgeons are required to practice procedures outside of the operating room. Simulation tools meet such demands. To ensure that such training results in improvement of surgical skills, it is necessary to be able to measure and assess surgeons’ skills. Thus, it is important to understand the performance differences between experienced and novice surgeons.

Previous studies distinguished experienced surgeons from novices using performance scores based on performance time, bleeding volume, and number of errors made during an operation. Some studies evaluated laparoscopic skills using objective evaluating methods such as the Global Operative Assessment of Laparoscopic Skills (GOALS) [1] and Global Evaluation Assessment of Robotic Skills (GEARS) [2].

Especially in laparoscopic surgery, some studies examined psychomotor skills. Using electromagnetic position-tracking sensors, kinematic analyses of motion, involving parameters such as time, path length, and speed, have been performed [3, 4]. Compared with the novices, experienced surgeons were expected to handle manipulators more smoothly during laparoscopic surgery and spend a shorter time to imagine their next action due to their experience, which would result in a shorter time without moving the forceps. However, quantitative parameters, such as motion smoothness (MS) and the blank time when the forceps are not moved, are controversial. There is no established objective method for evaluating targeting technique (applying forceps to an object), which is the basic movement of laparoscopic surgery. Most existing evaluation methods of motion smoothness use acceleration in the form of three-dimension vectors. We initially used the same method; however, it was difficult to distinguish between novices and experts. Therefore, we established the Short-Time Power of Difference (STPOD) method as an evaluation tool for assessing targeting technique. Herein we examined the effectiveness (construct validation) of the STPOD method as a new forceps kinematic analysis method.

## Materials and methods

**Fig. 1:**
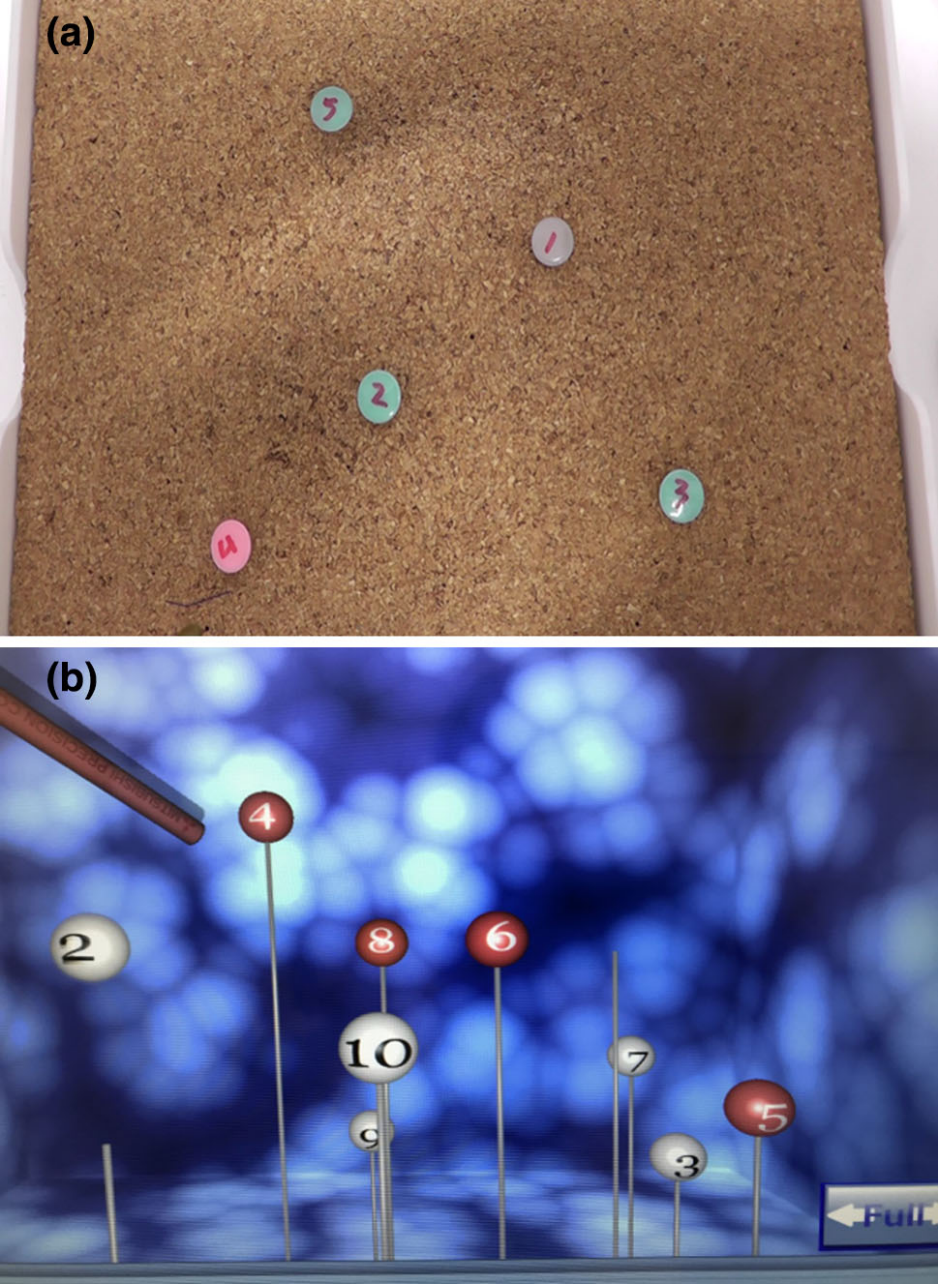
A graphical representation of the experiment. A box trainer with five numbered pins on a cork board. This task requires touching the pins with the tuppel in order. B targeting training image of LapPASS^®^. This task requires touching the red balloon with the right hand and the white balloon with the left in order

This study was approved by the Institutional Review Board of Yokohama City University. Participants signed their informed consent to participate in the study. The data gathered were coded, and all reporting was confidential and did not impact the official evaluation. Participants could choose to withdraw at any point during the study and they were made explicitly aware of this at the time of informed consent. Six residents were categorized as laparoscopic novices (no experience in laparoscopic surgery) and six urology doctors as laparoscopic experts (> 20 laparoscopic procedures completed and having a surgical skill qualification issued by the Japanese Society of Endourology) [5]. The dominant hand was all right. All participants were oriented to the box trainer and the training simulator LapPASS^®^ (Mitsubishi Precision, Japan, https://www.mpcnet.co.jp/product/lappass/) [6–9], and given a demonstration of both. The subjects in each category were then randomized into two groups using a randomizing program (https://en.calc-site.com/randoms/grouping). Group 1 underwent training with the box trainer followed by LapPASS^®^ and Group 2 with LapPASS^®^ followed by the box trainer (Fig. 1a and b).

The task in the box trainer was touching targets in order using forceps five times using each hand. One participant performed 10 times in total. The electromagnetic tracking system TrakSTAR^®^ (Mikimoto Beans, Japan, https://tracklab.com.au/products/brands/ndi/ascension-trakstar/) was used to acquire the position information of the forceps. The device was attached to the tip of the tuppel to get position information. The task in LapPASS^®^ consisted of participants performing hand-eye training six times. This hand-eye training consisted of applying the forceps to the targets in order.

**Fig. 2:**
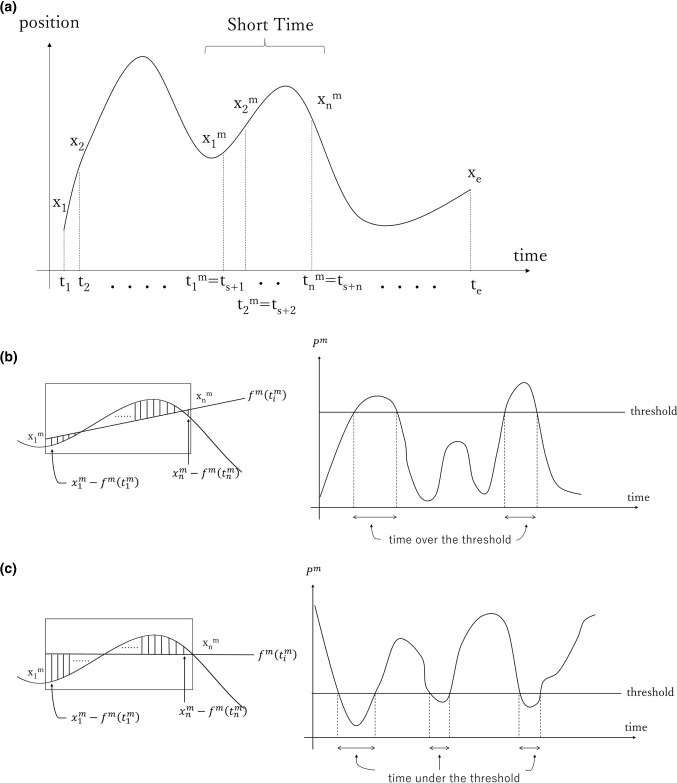
**a** A schematic diagram of the STPOD method. **b** A schematic diagram of power. Left image represents the calculation of $$x_{i}^{m} - f^{m} \left( {t_{i}^{m} } \right)$$. The box means the short time ‘m’ and $$f^{m} \left( {t_{i}^{m} } \right)$$ is the regression line ‘$$a^{m} t_{i}^{m} + b^{m}$$’. We calculate the Power ‘$$P^{m } $$’ using $$x_{i}^{m} - f^{m} \left( {t_{i}^{m} } \right)$$. Right image is a chronological representation of ‘$$P^{m}$$’. Time over threshold means ‘time not smooth.’ **c** A schematic diagram of blank time. In left image, $$f^{m} \left( {t_{i}^{m} } \right)$$ is average $$f^{m} \left( {t_{i}^{m} } \right)$$ is average ‘$$\overline{x}^{m} = \frac{1}{n}\mathop \sum \nolimits_{i = 1}^{n} x_{i}^{m}$$’. In right image, time under threshold means ‘time not moving’ (*i* start from 1 to n)

The STPOD method is entirely different from existing methods for kinematic analysis. STPOD is a way to evaluate and quantify “How much the distance is from the position of the tip of the tuppel either to the shortest path between the starting point and the end point, or to the average of all positions visited during a short time period.” The value obtained is denoted as $$P^{m}$$. We calculate STPOD (Power) and STPOD (Stop) making a graph with $$P^{m}$$ on the y-axis and time on the x-axis. When $$P^{m} $$ is less than the threshold, the surgical tool is considered as “not moving.” When $$P^{m}$$ is bigger than the threshold, the surgical tool is considered as “not linear nor smooth.” It was calculated using a simple product-sum operation and was suitable for real-time evaluation. A schematic diagram of the STPOD method is shown in Fig. 2a–c.

The following is an explanation of the STPOD method.

In the time series data $$\left[ {\left( {\begin{array}{*{20}c} {t_{1} } & {x_{1} } \\ \end{array} } \right),\left( {\begin{array}{*{20}c} {t_{2} } & {x_{2} } \\ \end{array} } \right), \ldots ,\left( {\begin{array}{*{20}c} {t_{e} } & {x_{e} } \\ \end{array} } \right)} \right]$$, extract short time period ‘m’ and define it as $$\left[ {\left( {\begin{array}{*{20}c} {t_{1}^{m} } & {x_{1}^{m} } \\ \end{array} } \right),\left( {\begin{array}{*{20}c} {t_{2}^{m} } & {x_{2}^{m} } \\ \end{array} } \right), \ldots ,\left( {\begin{array}{*{20}c} {t_{n}^{m} } & {x_{n}^{m} } \\ \end{array} } \right)} \right]$$ using superscript ‘m’. Here $t_{e}$ means time and $x_{e}$ means the position of the tip of the tuppel. $x_{e}$ represents a three-dimensional vector, but here is simplified to $x_{e}$ for clarity. Time period *m* means 0.5 [s] in the box trainer, and 1 [s] in the simulator. Next, we defined Power ‘$P^{m}$’ in this period as

1 $$ P^{m} = \mathop \sum \limits_{i = 1}^{n} \left\{ {\left( {x_{i}^{m} - f^{m} \left( {t_{i}^{m} } \right)} \right)\left( {0.5 - 0.5\cos \left( {2\pi \frac{{t_{i}^{m} - t_{1}^{m} }}{{t_{n}^{m} - t_{1}^{m} }}} \right)} \right)} \right\}^{2} $$

Here, $f^{m} \left( {t_{i}^{m} } \right)$ is a standard formula and is defined as average ‘$\overline{x}^{m} = \frac{1}{n}\mathop \sum \limits_{i = 1}^{n} x_{i}^{m}$’ or regression line ‘$a^{m} t_{i}^{m} + b^{m}$’.

*a*^*m*^ and *b*^*m*^ were defined as:

$$ \begin{aligned} a^{m} & = \frac{{\mathop \sum \nolimits_{i = 1}^{n} \left( {t_{i}^{m} - \overline{t}^{m} } \right)\left( {x_{i}^{m} - \overline{x}^{m} } \right)}}{{\mathop \sum \nolimits_{i = 1}^{n} \left( {t_{i}^{m} - \overline{t}^{m} } \right)^{2} }} \\ b^{m} & = \overline{x}^{m} - a^{m} \overline{t}^{m} \\ \end{aligned} $$

$\overline{x}^{m}$ and $\overline{t}^{m}$ were the averages of $x^{m} {\text{and}} t^{m}$, respectively.

The latter part of the formula $\left( {0.5 - 0.5\cos \left( {2\pi \frac{{t_{i}^{m} - t_{1}^{m} }}{{t_{n}^{m} - t_{1}^{m} }}} \right)} \right)$ is the Hamming window function.

In conclusion, formula (1) represents the sum of the squares of the distance from the position of the tip of the tuppel either to the shortest path between the starting point and the end point, or to the average of all positions visited during time period *m*, given weight by the window function.

There are two evaluation indexes by the STPOD method: ‘power,’ which indicates wasteful movement, and ‘blank time’, which indicates when movement is stopped.

### [Evaluation item 1: power]

We used the regression line as the standard. When ‘$P^{m}$’ is bigger than the threshold, the surgical tool is considered to be “not linear.” We calculated the sum of the time sections above the threshold. A smaller value means that the surgical instrument moves linearly and smoothly (Fig. 2b).

### [Evaluation item 2: blank time]

We used the average as the standard. When ‘$P^{m}$’ was less than the threshold value, the surgical tool is considered to be “stop.” We calculated the sum of the time sections below the threshold. A smaller value means that there was shorter stagnation of movement (Fig. 2c).

We evaluated “time” and “distance” as conventional comparative values.

Statistical analysis was performed using EZR [10], which is a graphical user interface for R (The R Foundation for Statistical Computing, Vienna, Austria). Mann–Whitney *U* test was performed for each pair of groups. In order to correct for multiplicity, we performed the Bonferroni correction. As a result, the significance level of the box trainer (*n* = 4) changed from* p* < 0.05 to *p* < 0.0125 (0.05/4 = 0.0125), and of the simulator (*n* = 5) changed to *p* < 0.01 (0.05/5 = 0.01).

## Results

**Fig. 3:**
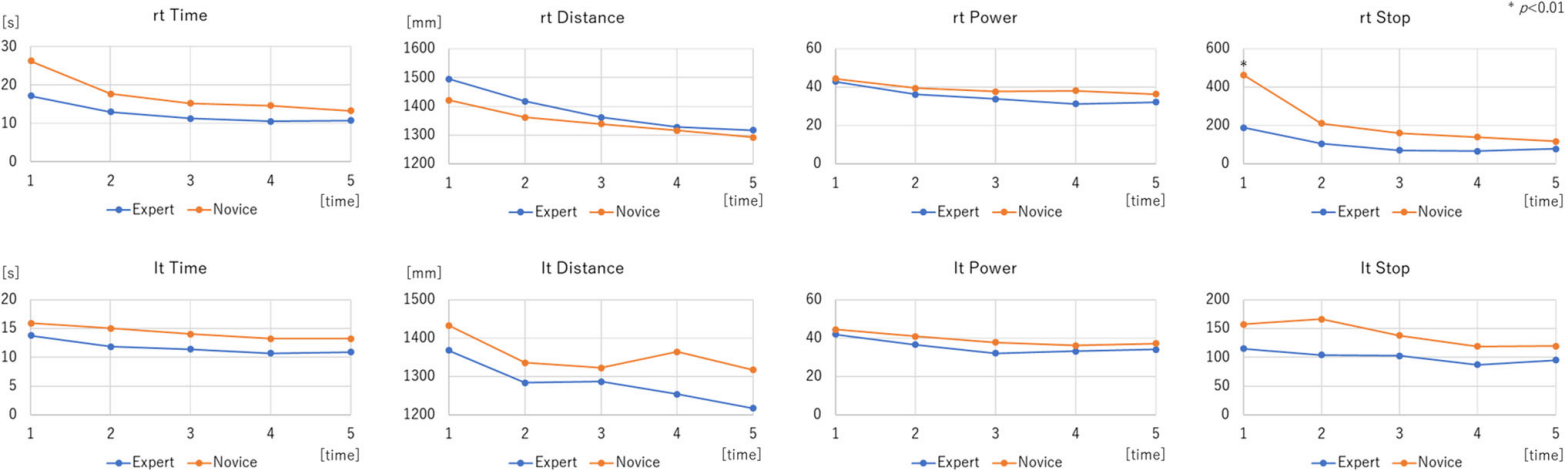
Learning curve of box trainer between the expert and novice groups. “rt” stands for right hand and “lt” for left hand

**Fig. 4:**
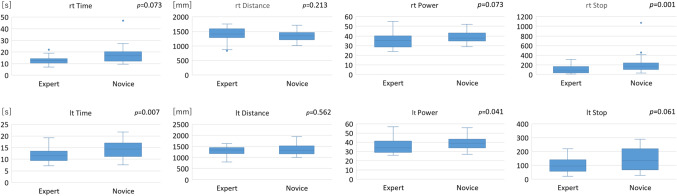
Comparison of the box trainer at all times between the expert and novice groups. The results are presented as box and whisker plots, in which every box has a line at every quartile, median, and upper quartile value. The whiskers are presented as lines that extend from each end of the box to show the extent of the remaining data. Outliners are plotted with circles

In the box trainer, there was a tendency for all the parameters to improve in both groups as the number of times increased (Fig. 3). For all repetitions together, STPOD (Stop) on the right side and time on the left side of the experts were significantly lower than those of the novices. This shows the construct validation of STPOD (Stop) in the box trainer (Fig. 4) (Table 1).

**Table 1:** Results of the Box trainer task between Expert and Novice groups

	Playtime (s)		*p*	Distance (mm)		*p*	Power		*p*	Stop		*p*
	Expert	Novice		Expert	Novice		Expert	Novice		Expert	Novice	
*Right*												
Time												
1	17.2 ± 2.94	26.3 ± 9.68	0.026	1495 ± 274	1422 ± 182	0.240	43.0 ± 6.02	44.5 ± 5.35	0.629	190 ± 60	464 ± 286	0.009
2	13.1 ± 2.28	17.7 ± 4.3	0.180	1417 ± 267	1362 ± 168	0.589	36.2 ± 3.48	39.5 ± 5.28	0.329	106 ± 75	212 ± 103	0.065
3	11.3 ± 2.09	15.2 ± 3.88	0.132	1362 ± 226	1339 ± 214	0.589	33.8 ± 6.81	37.7 ± 6.94	0.520	72 ± 55	161 ± 66	0.065
4	10.6 ± 2.15	14.6 ± 3.57	0.132	1328 ± 255	1316 ± 134	0.818	31.3 ± 5.52	38.2 ± 5.43	0.107	67 ± 60	140 ± 60	0.132
5	10.8 ± 2.73	13.3 ± 2.95	0.240	1317 ± 233	1293 ± 179	0.310	32.1 ± 5.58	36.3 ± 4.82	0.297	79 ± 73	118 ± 61	0.378
Average	12,6 ± 2.12	17.4 ± 7.17	0.132	1384 ± 240	1346 ± 183	0.589	35.3 ± 5.02	39.2 ± 6.28	0.336	103 ± 51	219 ± 192	0.015
*Left*												
Time												
1	13.8 ± 3.40	15.9 ± 3.82	0.394	1368 ± 258	1432 ± 87	0.937	42.0 ± 9.7	44.5 ± 5.7	0.936	114 ± 55	157 ± 82	0.423
2	11.9 ± 2.66	15.0 ± 3.86	0.240	1283 ± 205	1335 ± 211	0.937	36.7 ± 7.6	41 ± 8.3	0.575	104 ± 49	166 ± 88	0.240
3	11.4 ± 2.35	14.0 ± 3.48	0.180	1286 ± 218	1322 ± 192	0.818	32.2 ± 4.9	37.8 ± 4.7	0.146	103 ± 52	138 ± 74	0.589
4	11.8 ± 2.55	13.3 ± 3.42	0.180	1254 ± 263	1364 ± 316	0.818	33.1 ± 6.3	36.1 ± 6.4	0.465	87 ± 52	119 ± 58	0.336
5	10.9 ± 1.86	13.2 ± 3.62	0.394	1217 ± 215	1317 ± 214	0.818	34 ± 5.7	37.2 ± 6.1	0.520	95 ± 42	119 ± 64	0.699
Average	11.7 ± 2.84	14.3 ± 3.79	0.394	1282 ± 238	1354 ± 221	1.000	35.6 ± 7.9	39.3 ± 7.1	0.310	101 ± 51	140 ± 77	0.485

**Fig. 5:**
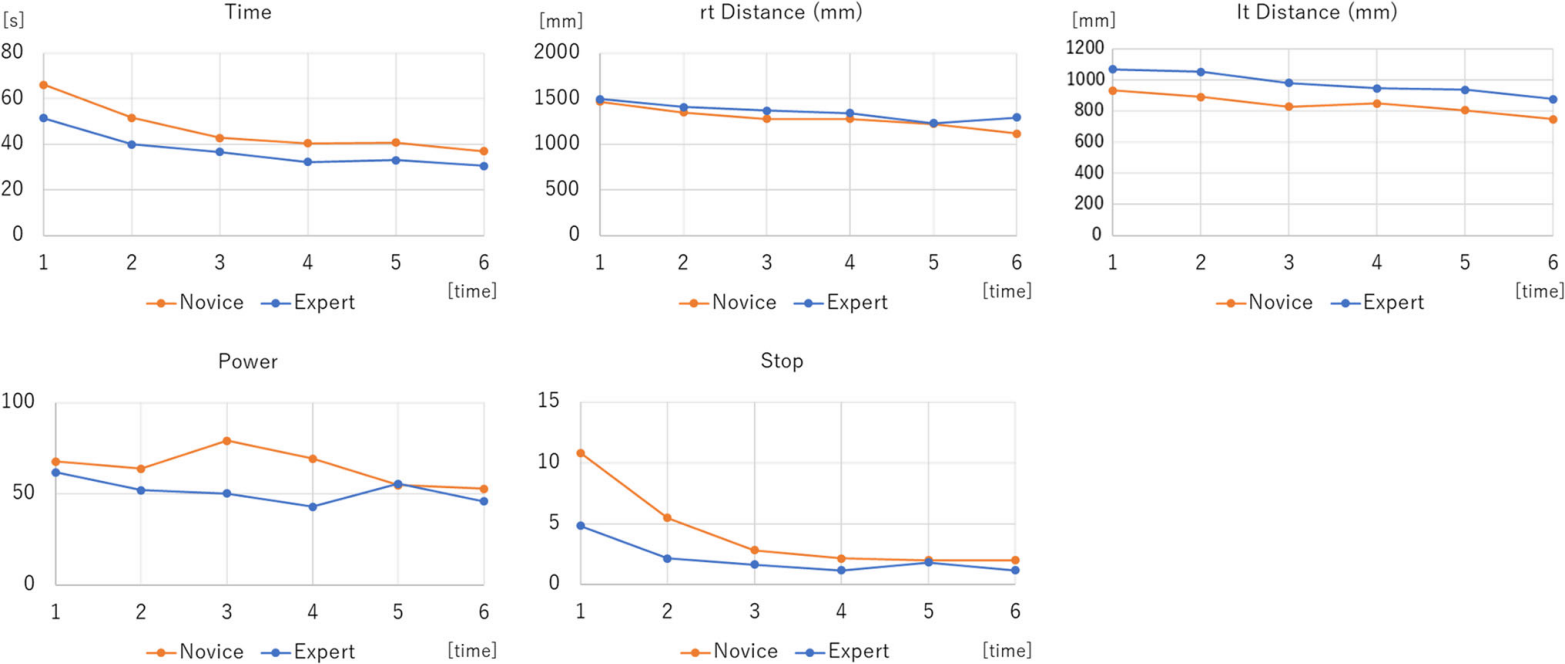
Learning curve of the simulator between the expert and novice groups. “rt” stands for right hand and “lt” for left hand

In the simulator, both groups improved their score as the number of times increased (Fig. 5).

**Fig. 6:**
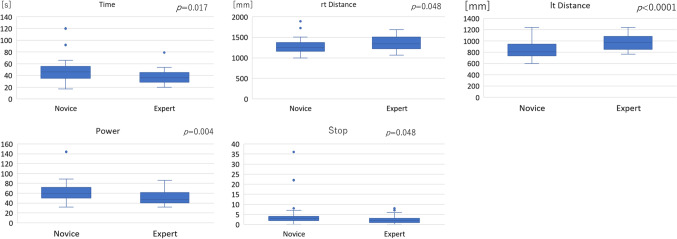
Comparison of box trainer at all times between the expert and novice groups. The results are presented as box and whisker plots, in which every box has a line at every quartile, median, and upper quartile value. The whiskers are presented as lines that extend from each end of the box to show the extent of the remaining data. Outliners are plotted with circles

For all repetitions together, there was a significant difference in distance on the left side and STPOD (Power) between the experts and the novices. This shows the construct validation of STPOD (Power) in the simulator (Fig. 6) (Table 2).

**Table 2:** Results of the simulator task between Expert and Novice groups

	Playtime (s)		*p*	rt Distance (mm)		*p*	lt Distance (mm)		*p*	Power		*p*	Stop		*p*
	Expert	Novice		Expert	Novice		Expert	Novice		Expert	Novice		Expert	Novice	
*Time*															
1	51.5 ± 14.5	66.1 ± 25.5	0.485	1498 ± 150	1469 ± 262	0.699	1068 ± 61.2	932 ± 197	0.240	4.83 ± 2.4	10.8 ± 11	0.580	62 ± 18	68 ± 15	0.810
2	40 ± 8.35	51.7 ± 20.3	0.375	1410 ± 156	1352 ± 128	0.699	1053 ± 92.3	891 ± 155	0.093	2.17 ± 0.90	5.5 ± 7.4	1.000	52 ± 12	64 ± 15	0.297
3	36.7 ± 9.14	42.8 ± 11.7	0.377	1372 ± 149	1282 ± 134	0.394	981 ± 137	828 ± 117	0.093	1.67 ± 1.1	2.8 ± 2.5	0.564	50 ± 14	79 ± 31	0.170
4	32.3 ± 6.21	40.5 ± 11.8	0.394	1343 ± 151	1281 ± 136	0.589	946 ± 82.1	849 ± 152	0.240	1.17 ± 0.90	2.2 ± 1.2	0.168	43 ± 6.3	69 ± 37	0.198
5	33.2 ± 6.89	40.8 ± 13.0	0.422	1234 ± 125	1225 ± 79	0.937	935 ± 150	805 ± 110	0.394	1.83 ± 0.90	2 ± 1	0.738	56 ± 16	55 ± 9.6	0.810
6	30.7 ± 7.06	37 ± 10.3	0.261	1298 ± 156	1117 ± 60	0.026	877 ± 114	748 ± 70	0.132	1.17 ± 0.90	2 ± 1.2	0.214	46 ± 9.6	53 ± 10	0.394
Average	37.4 ± 11.5	46.5 ± 19.1	0.240	1359 ± 170	1288 ± 183	0.240	977 ± 129	842 ± 151	0.132	2.14 ± 1.81	4.2 ± 6.5	0.147	51 ± 15	65 ± 24	0.065

**Fig. 7:**
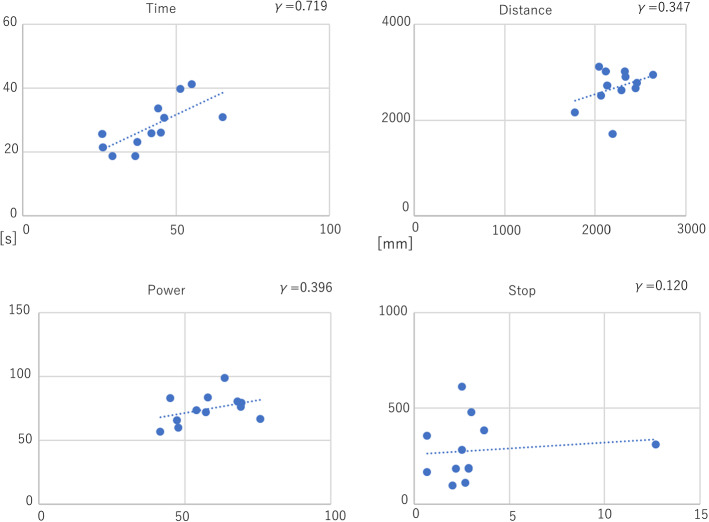
Pearson correlation plot of box trainer versus simulator in the expert and novice groups

By evaluating the correlation of parameters between the box trainer and the simulator, time showed the strongest correlation (*γ* = 0.719). Next, STPOD (Power) and distance showed a mild correlation (*γ* = 0.396 and *γ* = 0.347, respectively) (Fig. 7).

## Discussion

Conventionally, for the evaluation of the simulator, time, the amount of bleeding, and objective evaluations, such as GOALS and GEARS, have been used. When the surgeon performed the procedure carefully and spent a lot of time, the amount of bleeding was small and the results were often good; however, it was expected that the burden on the patient would be large. Previous studies reported that a shorter operation time lead to a reduction in postoperative complications [11–14]. Ideally, a smooth and short surgery is required; however, it has been difficult to evaluate smoothness. In general, it is predicted that the more experienced surgeons have smoother motions and obtain lower values for MS compared to less experienced ones. Furthermore, it is predicted that the experts need a shorter time to think about what they should do next and this results in a shorter blank time.

As an evaluation method, a scoring system, such as GOALS and GEARS, is used; however, these evaluation methods depend on the surgical expert’s subjective decisions. For this reason, studies were conducted to distinguish MS between experts and novices using objective methods; however, this was challenging. Maithel et al. evaluated MS using the Computer-enhanced Laparoscopic Training System [15], and found no significant difference. Hofstad et al. evaluated psychomotor skills using the D-Box Basic Simulator [16]. The Aurora Electromagnetic Measurement System was used for the tracking of the forceps and MS was defined as a total change in acceleration of the tip of the instrument. There was a significant difference only in the non-dominant hand when comparing novices to experts and intermediates. A further study assessed cholecystectomy performed in a porcine liver box model [17], and MS was defined the same as Hofstad et al.; however, there was no significant difference between the groups. On the other hand, Escamirosa et al. evaluated surgical skills by 13 motion analysis parameters using the EndoViS Training System [18]. The motion of the forceps was recorded by a video-tracking system. They defined MS as abrupt changes in acceleration resulting in jerky movements of the instrument (m/s^3^). There was a significant difference between the experts and novices in all three tasks, peg transfer, pattern cutting, and intracorporeal knot suture. We initially used acceleration to evaluate MS using existing methods; however, it could not distinguish between the novices and experts. On the other hand, STPOD was able to distinguish the two groups. Thus, this new method is an alternative way to evaluate MS.

In the present study, right-hand distance of the box trainer in the expert group was longer than that of the novice group. This may reflect the habits of each surgeon. In actual laparoscopic surgery, when moving forceps between two points, the forceps are not moved in a straight line and are often moved after the forceps have been pulled back to the hand. Because of their real-world experience, experts may take a longer distance. This study was conducted in the order of right to left hand; thus, the participants recognized the position of the pins and became familiar with the movement in the right-hand session, and this may have resulted in the lack of a significant difference between the two groups in the left-hand session.

The most suitable objective method to evaluate blank time is controversial. Uemura et al. evaluated forceps movements using a labeling system based on predefined terminology. They concluded that skilled participants had a shorter blank time (time without forceps movement) than novices. They proposed that the time spent holding the forceps but not moving them was time spent thinking, and that the shorter blank time for the skilled users was due to their experience and stable manipulation movements [19]. In addition, Hofstad et al. reported that there was a significant difference in the idle percentage (percentage of total time the instrument is moved at speed < 2 mm/s) [17]. On the other hand, some reports concluded that there were no significant differences between expert and novices when defining idle time as the percentage of time where the instrument is considered to be still [4, 18].

By using the STPOD method, we evaluated the degree of flicker of the forceps and the blank time when the forceps were not moved. We predicted that there would be a little flicker and blank time in the experts’ surgery.

In this study, we found that the experts had less flicker and a shorter blank time in both the box and the simulator. Furthermore, although it is inferior to the objective evaluation method “time” that has been used for a long time, we showed a correlation of STPOD between the box and the simulator. Thus, the STPOD method is effective for evaluating forceps dynamics.

There are some limitations in this study. First, the number of subjects was limited. Second, we focused only on targeting and the task that the participants performed was just moving the forceps between fixed targets. In an actual surgery, there are much more complex motions. For this reason, we did not use GOALS evaluation. In the future, evaluation in an environment closer to actual surgery and comparing the STPOD method with other methods, such as GOALS, are necessary. Third, a comparison between our proposal and existing motion analysis parameters was not performed. Comparing STPOD with the existing method to evaluate motion smoothness is necessary.
